# Relevance of Variations in the Opposing Dentition for the Functionality of Fixed and Removable Partial Dentures: A Systematic Review

**DOI:** 10.1155/2012/876023

**Published:** 2012-11-04

**Authors:** Bernhard Pommer, Martin Krainhöfner, Georg Watzek, Gabor Tepper, Charalabos-Markos Dintsios

**Affiliations:** ^1^Department of Oral Surgery, Bernhard Gottlieb School of Dentistry, Medical University of Vienna, Sensengasse 2a, 1090 Vienna, Austria; ^2^Department of Prosthodontics, Bernhard Gottlieb School of Dentistry, Medical University of Vienna, Sensengasse 2a, 1090 Vienna, Austria; ^3^Department of Public Health, Faculty of Medicine, Heinrich Heine University Düsseldorf, Moorenstr. 5, 40225 Düsseldorf, Germany; ^4^Division of Market and Reimbursement, HTA and Health Economic Evaluation, Research-Based Pharmaceutical Companies (vfa), Hausvogteiplatz 13, 10117 Berlin, Germany

## Abstract

The aim of this systematic review was to evaluate the functionality of fixed and removable partial dentures as test interventions in relation to variations in the opposing dentition and their prosthetic restoration. The abstracts identified in the respective databases were screened independently by two investigators. RCTs and uncontrolled studies were considered, provided the patients were included consecutively and the confounding variables were adequately monitored. Seventeen papers were included. The study and publication quality was assessed using a “biometric quality” tool showing an overall poor quality. The reported outcomes, such as survival rates, were in each case obtained from a single study. Two possible trends could be deduced for the endpoint longevity: (a) the first trend in favor of removable partial dentures, compared to fixed partial dentures, with a fully edentulous opposing arch fitted with a removable prosthesis; (b) the second trend in favor of implant-supported partial dentures, compared to conventionally fixed partial dentures, with natural opposing dentition or with a removable partial denture in the opposing arch. No evidence could be generated as to whether, and if so how, variations in the opposing dentition have a bearing on the decision to fit a partially edentulous arch with a fixed or removable partial denture.

## 1. Introduction

The German decision maker Federal Joint Committee (FJC) commissioned the Institute for Quality and Efficiency in Health Care (IQWiG) to perform a scientific evaluation on the relevance of variations in the opposing dentition for the functionality of fixed and removable partial dentures. On May 20, 2010 the FJC decided, on the basis of the present systematic review, that routine care cannot be made dependent upon whether the opposing arch is provided with fixed or removable partial dentures [1] and has therefore revoked its previous directive on fixed allowances for health care services.

In the Fourth German Dental Oral Health Study (DMS-IV) it was estimated that, without taking wisdom teeth into account, in persons aged between 35 and 44 years, on average about 2.7 teeth were missing; 48.5% of missing teeth had been replaced [2]. In persons aged between 65 and 74 years, 14.2 teeth on average were missing and 31.3% had an edentulous upper arch; 88.7% of missing teeth had been replaced.

With the exception of a shortened dental arch, every tooth gap requires the earliest possible prosthetic restoration in order to avoid secondary damage. When deciding upon the type of denture most suitable for the respective patient, besides the nature of the opposing dentition or the type of prosthetic restoration in the opposing arch [3], the following factors play a crucial role: (1) size of gap (number of missing teeth), type of gap (free-end or interdental), and gap localization [4]; (2) age of patient [5]; (3) specific factors in the patient's lifestyle, including in particular the quality of the patient's oral hygiene as well as his or her tobacco consumption [6]. 

The subsequent specifications define the aim of the investigation, which is as follows: assessment of the functionality of fixed and removable partial dentures as test interventions in relation to variations in the opposing dentition and their prosthetic restoration. Following patient-relevant outcomes were evaluated: (1) “denture longevity” [7], (2) “change in dietary habits” [8], (3) “oral health-related quality of life” [9] to include patient satisfaction and phonetic rehabilitation, which are parameters relevant to quality of life, and (4) “denture cleansability and aftercare required” [10].

## 2. Material and Methods 

The test intervention assessed was the treatment of residual dentition by means of fixed partial dentures depending on the nature of the opposing dentition. The comparator intervention assessed was the treatment of residual dentition by means of removable partial dentures or combined fixed/removable partial dentures, again depending on the nature of the opposing dentition. Implant-supported dentures (fixed or a combination of fixed/removable partial dentures) were also considered in the assessment of the test and comparator interventions. Control groups receiving no treatment at all were not considered.

Adult patients with residual dentition who had an indication for restoration with partial dentures were included in the investigation. Residual dentition was classified as interdental (Kennedy Class III+IV) as well as free-end gaps (Kennedy Class I+II) [11] comprising at least the first two molars. All patients with the following conditions were excluded from the investigation: posttraumatic status, status after carcinoma resection, craniofacial deformities and syndromes, and oligodontia. 

Systematic literature searches were carried out in the following databases: CENTRAL, MEDLINE, EMBASE, BIOSIS, SciSearch, CCMed, DARE, and HTA (search period from 1982 to 2009, inclusive). In addition, a manual search was carried out in German dental journals and the search was extended topic specifically to the following databases: CDSR, CDMR, CDMS, NHS EED, CINAHL, AMED, CAB abstracts, GLOBAL Health, ISTPB + ISTP/ISSHP, Medikat, and the publisher databases of Karger (secondary search), Kluwer, Springer, Thieme, and Hogrefe (secondary search). Finally, the opportunity to cite additional topic-relevant studies for the timespan until middle of 2008 was provided in July 2008 when interested parties were invited to submit written comments on a preliminary version of the report.

Randomized controlled trials (RCTs) together with prospective and retrospective studies without control groups were included for the patient-relevant outcomes, provided the patients were included in the study consecutively and the confounding variables were adequately monitored. Also included in the assessment were case reports and case series with a sample size of at least ten and of adequate biometric quality to avoid any bias in selection. The minimum observation time for all studies was a follow-up period of six months.

The literature screening was carried out by two reviewers independently of each other. After assessing the biometric quality of the studies, the results from the individual studies were collated according to therapy goals and outcomes, compared, and described. IQWiG's preliminary benefit assessment, the preliminary report, was published on the Internet in German language and interested parties invited to submit written comments (https://www.iqwig.de/download/N05-02_Vorbericht_V_1_0_Relevanz_der_Beschaffenheit_der_Gegenbezahnung.pdf).

In addition to data consistency within the publication, particular attention was paid to the homogeneity of the follow-up period. In prospective studies, this is equivalent to homogeneity in the age of the prostheses fitted. This factor was also examined in the case of retrospective analyses, that is, whether the age of the prostheses deviated considerably from each other or at least whether their age was adequately documented in the studies. In studies using questionnaires, particular focus was placed on the methodology of patient recruitment, paying special attention to the application of a consecutive procedure.

The complexity of the topic lies in the great variability of very different dental patterns resulting from the lack of one or more of the total of 32 teeth in the human dentition. To enable subgroup analyses of patients with the numerous teeth patterns, documentation of gap classification according to Kennedy [11] was a major factor in the usability of the individual results for the present systematic review.

Finally, taking the above-mentioned aspects into account, the study and publication quality was assessed by means of a “biometric quality” tool comprising four grades (no detectable flaws, minor flaws, major flaws, and unclear). 

The grades were defined a priori as follows: “minor flaws” exist if it is assumed that their correction would not substantially influence the results and therefore the overall conclusions of the study. In the case of “major flaws” the overall conclusions of the study would be called in question, as correction of the flaws might lead to different conclusions. 

## 3. Results

**Figure 1:**
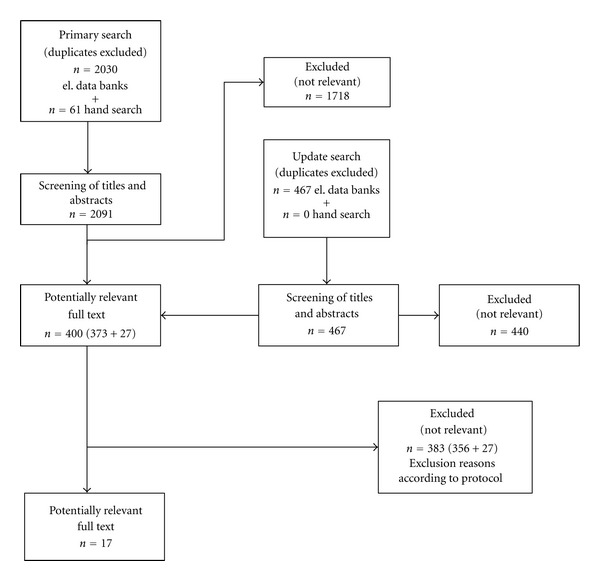
Flow chart literature search.

Initially, a total of twenty five papers were identified that met the inclusion criteria. After more detailed screening, eight studies had to be excluded from the assessment, as the data provided could not be broken up on the basis of variations in the opposing dentition (Figure 1). In five out of 17 papers definitely included, there were prepublications with no additional relevant information. In eight of the 17 studies, there was information about the “denture longevity” outcome, in five studies information on the “change in dietary habits” outcome, in four studies information on the “oral health-related Quality of life” and “patient satisfaction” outcome, and in nine studies information on the “denture cleansability and aftercare required” outcome, whereby 11 studies reported on one outcome, three studies on two, and three studies on three outcomes. Five publications reported only on fixed partial dentures, three publications only on removable partial dentures, one publication on fixed and removable partial dentures, one publication on fixed partial dentures and complete dentures, three publications on removable partial dentures and complete dentures, one publication on removable partial dentures and fully dentulous patients, and three publications on removable partial dentures and complete dentures as well as fully dentulous patients. There were control interventions in nine papers; however, in eight cases they represented interventions that did not meet the inclusion criteria (i.e., complete dentures or fully dentulous patients) (see Tables 1 and 2). This ultimately resulted in indirect comparisons being carried out on the test interventions of fixed versus removable partial dentures in relation to variations in the opposing dentition.

The overall study and publication quality of the relevant studies was for the most part inadequate (Table 3). There was only one prospective trial on the topic under investigation [12] that could be described as randomized controlled, yet it provided no information on the randomization technique used. The six prospective studies [13–18] identified revealed unequal periods of observation and flaws in how study discontinuations were dealt with. The three retrospective studies [19–21] also revealed considerable flaws in the quality of studies and publication. The findings were similar for the seven prevalence studies identified [22–28], although here it was mainly the selection methods of the patient population that were inadequately described. 

### 3.1. “Denture Longevity” Outcome

Of the eight studies identified for the “denture longevity” outcome, one had no detectable flaws, two had minor, and five had major flaws in the biometric quality of the studies and publication. A comparison between the longevity of fixed and removable partial dentures was only possible for one opposing dentition variant (complete dentures in the opposing arch) (Tables 4 and 5).

Only one trial [12], described as randomized controlled, contains data on the longevity of fixed and removable partial dentures in combination with a complete denture in the opposing arch. The validity of this trial is reduced through the following biometric flaws: (1) no data on the Kennedy classes of the intervention arches; (2) incomplete data on prognostic factors and comorbidity; (3) inhomogeneous size of tooth gaps in the group with fixed partial denture: 44.4% teeth gaps of two to three teeth, 25.9% gaps of four to five teeth, and 29.7% gaps of nine to 11 teeth; (4) no data on teeth gaps for the group with removable partial denture; (5) drop-out rate of 18.9% over a follow-up period of five years; (6) detailed description of randomization procedure is missing (only mentioned as a term), so it should rather be classed as a nonrandomized controlled trial; (7) no significance level given (*P* value) in the subgroup analysis. Consequently, a significant difference cannot be considered as detected in the 5-year survival rate of fixed partial dentures (95.2%) and removable partial dentures (100%) with a complete denture in the opposing arch. 

Data on the longevity of fixed tooth-borne partial dentures with differing variations in the opposing dentition were found in two studies: in one publication [15], the 4-year survival rate with natural opposing dentition is given as 86,7%; in the above-mentioned RCT [12], the 5-year survival rate with complete denture in the opposing arch is given as 95.2%. Apart from other differences in the study design and setting, a direct comparison between these two trials is limited by the different follow-up periods, since one of the publications gives no survival rates for shorter periods.

Data on the longevity of fixed implant-supported partial dentures with differing variations in the opposing dentition were found in two additional trials: the 3-year survival rate in case of natural opposing dentition is given as 97.8% in one publication [13]; the survival rate for fixed implant-supported partial dentures in the opposing arch after an average followup of 44.5 months is given as 100% in the other publication [16]. Apart from other differences in the study design and setting, a direct comparison of these data does not appear to have much value, since the follow-up period in one of the studies does not indicate survival rates for shorter periods.

No conclusions can be drawn on the basis of the existing data regarding whether variations in the opposing dentition influence the longevity of fixed or removable partial dentures (Table 6). Only two trends can be deduced. (a) The first trend suggests increased longevity of removable partial dentures, compared to fixed partial dentures, with a fully edentulous opposing arch fitted with a removable prosthesis. (b) The second weaker trend suggests increased longevity of implant-supported partial dentures, compared to conventionally fixed partial dentures, with natural opposing dentition or with a removable partial denture in the opposing arch.

### 3.2. “Dietary Habits” Outcome

There were major flaws in the quality of the biometric studies and publication in all 5 studies that contained data on the change in dietary habits in fixed and removable partial dentures in relation to variations in the opposing dentition. As no evaluable data were found on dietary habits in patients fitted with a fixed partial denture, it was not possible to compare with dietary habits in patients fitted with a removable partial denture (Tables 7 and 8). 

Data on the relevance of opposing dentition for removable partial dentures could only be drawn from one trial [17]. The validity of this trial is reduced due to the following biometric flaws: (1) inhomogeneous residual dentition in the intervention/opposing arch: on average 17.4 teeth in the opposing natural dentition group, 11.8 teeth in the opposing removable partial denture group, and 5 teeth in the opposing complete denture group; (2) no data on the Kennedy classes in the intervention arches; (3) no data on prognostic factors or on comorbidity; (4) only male patients between 67 and 68 years of age; (5) inhomogeneous age of prostheses: 35% less than 2 years old, 48% between 2 and 9 years old, and 17% over 10 years old; (6) data collection instrument based on 6 hard and 6 soft meals not validated; (7) inappropriate percentage analysis of restriction in dietary habits. In another trial, all patients interviewed featured natural dentition in the opposing arch, so that different dentition patterns could not be compared.

The existing data does not allow any conclusion to be drawn on whether variations in the opposing dentition influence dietary habits when a fixed or removable partial denture is fitted. The data in another trial [25] show that, in the case of removable partial dentures only, no or little difference can be determined in relation to the opposing dentition when eating soft or hard food (Table 9).

### 3.3. “Quality of Life and Patient Satisfaction” Outcome

Out of the four identified trials, one [12] displayed minor flaws and three [20, 22, 24] major flaws in the biometric quality of the studies. It was only possible to compare the satisfaction between a fixed and removable partial denture with one opposing dentition variant (complete denture in opposing arch).

One trial described as randomized controlled [12] contained data on patient satisfaction for fixed and removable partial dentures in combination with a complete denture in the opposing arch. Due to the biometric flaws already described in the results for the “denture longevity” outcome, this trial should be designated nonrandomized. A significant difference (*P* < 0.05) is indicated in patient satisfaction regarding stability in general and during chewing with fixed (77.8% and 85.2% of patients, resp., were satisfied) and with removable partial denture (61.5% and 53.9%, resp.). This effect cannot be viewed as proven due to the biometric flaws. However, the fact that fixed prostheses have greater stability than removable ones appears reasonable.

Data on general patient satisfaction with removable partial dentures and differing variations in the opposing dentition were found in one trial [22]: the percentage of satisfied patients with removable partial denture in the opposing arch was 37% (*n* = 102), and 65% for those with complete denture in the opposing arch (*n* = 147). However, due to the variable sampling size and the unequal age of prosthesis (one to 15 years), a comparison of these data would not appear to be worthwhile.

Based on the existing data, it is not possible to draw any conclusions on whether variations in the opposing dentition influence patient satisfaction when fitting fixed or removable partial dentures.

### 3.4. “Cleansability and Aftercare Required” Outcome

Data on denture cleansability and aftercare required when fitting fixed and removable partial dentures were included in nine publications. Of these trials, two showed minor [12, 13] and seven major flaws [20–24, 27, 28] in the biometric quality of studies and publication. It was only possible to compare the maintenance requirements of fixed and removable partial dentures for one opposing dentition variant (complete denture in the opposing arch). As no analysable data were found on prosthesis cleansability or aftercare required in fixed partial dentures, it was not possible to make a comparison with the prosthesis cleansability or aftercare required in removable partial dentures.

Only one RCT [12] contained data on the maintenance requirements of fixed and removable partial dentures when the opposing arch was fitted with a complete denture. However, as already mentioned above, it contained biometric flaws that reduced its validity. Consequently, a significant difference could not be proven in the level of repair required for fixed partial dentures (22.2% of prostheses) and removable partial dentures (23%) in combination with a complete denture in the opposing arch.

In another trial [21], data were found on the level of repair required for removable partial dentures with differing variations in the opposing dentition: the number of repairs required within a period of 16 months was 72 for natural opposing dentition, eight for removable partial dentures in the opposing arch, and 18 for removable complete dentures in the opposing arch. However, due to the nondocumented sample size of the individual subgroups and the inhomogeneous age of prosthesis (one to six years), a comparison of these data would not appear to be worthwhile.

No conclusions could be drawn on the basis of the existing data as to whether variations in the opposing dentition influence denture cleansability and aftercare in case of a fixed or removable partial denture.

## 4. Discussion

After a comprehensive literature search and an assessment of the evidence base on the relevance of variations in the opposing dentition for the functionality of fixed and removable partial dentures, no robust conclusions could be drawn. This is due both to the low quantity and the methodological weaknesses of the few studies identified. Only one of the 17 studies compared the two denture types investigated within the context of a controlled prospective study, even though this study cannot be regarded as an RCT either, due to its inadequately documented randomization procedure. All the remaining data extracted originated from prospectively or retrospectively planned studies without a control group (one-arm studies) or from one-off data collections by way of questionnaires. It should be noted, however, that all the studies included were not per se concerned with the question of the relevance of opposing dentition but addressed this question mostly in the form of subgroup analyses. For this reason the literature search for this report was particularly time intensive, as it could mostly be decided only on the basis of full-text screening whether a subgroup analysis considering opposing dentition had been performed. 

Within the framework of the quality assessment for the present systematic review, the serious biometric flaws in study and publication quality identified in nearly all studies refer to the research question investigated here, that is, the relevance of opposing dentition. This quality assessment is not an evaluation of the informative value of the individual studies with regard to their original research questions. 

### 4.1. Denture Longevity

The assessment of denture longevity, that is, the survival or success rate of dentures, should follow a clear definition. It must be quite evident what medical, functional, or even aesthetic requirements the dentures must fulfill in order to be classed as “functioning” or “successful.” In the present systematic review all irreparable damage was viewed as a failure (end of denture longevity) and all repairable damage was allocated to the outcome “Denture aftercare required.”

Functional and medical reasons (medical failure = tooth loss) account for 69.5% and 28.5% of fixed partial dentures failures, respectively [31]; removable dentures fail nearly exclusively for medical reasons [32]. Implant-supported fixed dentures can fail due to prosthesis failure but also through failure of the implants themselves [33]. In most of the studies included, no definition was offered as to what criteria were applied for success of the dentures, nor was the status of the opposing dentition over the course of the entire study period clearly reported. 

### 4.2. Change in Dietary Habits

Standardized procedures should also be defined for the assessment of dietary habits. First, some differentiations should be made between the objective chewing performance (i.e., the instrumentally measured ability to break down a specific test meal into pieces) and subjective chewing efficiency (i.e., chewing capacity as experienced by the patient), depending on the status of the opposing dentition. Both parameters diminish linearly with the number of missing teeth [34]. Furthermore, different, nonstandardized test meals have been used in various studies [9].

Some objective tests indicate that by means of fixed and removable dentures the chewing function can be equally restored [35], whereas others claim a better chewing capacity for hard food with fixed prostheses [36]. However, it is still controversial whether the objective effect measured correlates with the patient's subjective perception. Objectively measured improvements in chewing capacity through adaptation of the prosthesis have not been perceived by patients in previous studies [37]. A study in which patients with only one partial prosthesis that estimated their chewing ability to be the same as that of patients with two complete prostheses shows that the parameter “subjectively reported chewing efficiency” is potentially flawed [38]. Independent of the choice of measurement methods, changes in dietary habits should always be compared intraindividually.

A further patient-relevant aspect is the change in the perception of taste through dentures. However, this affects only removable prostheses in the upper jaw. Studies were able to show that dissatisfaction due to a changed perception in taste was evident only for prostheses covering the entire palate [39], that is, for complete prostheses. This aspect plays no role for the removable partial prostheses assessed in this systematic review.

### 4.3. Oral Health-Related Quality of Life and Patient Satisfaction

Oral health-related quality of life was not analyzed in the studies identified; we therefore extended the outcomes investigated and also included patient satisfaction so as to be able, at least, to draw conclusions on this quality-of-life relevant parameter. Patient satisfaction with fixed or removable dentures should always be compared intraindividually. Every patient should first be provided with removable dentures and then, after assessment of satisfaction, with fixed ones. This approach is more costly than an interindividual assessment, but reduces uncertainty and contradictions in patients' statements. As an inverse approach is not feasible on practical grounds, an intraindividual approach would, however, have to dispense with randomization with regard to the sequence of the interventions, as the study would not have a cross-over design in the proper sense of the term. Particularly in view of the variety of patterns regarding the status of teeth and opposing dentition, this study design represents the most reliable comparison between fixed and removable dentures for the evaluation of patient satisfaction. This design was not found in any of the studies included.

Independently of the nature of the opposing dentition, patient satisfaction has been found to be 96% for fixed dentures [40] and 90% for removable ones [41]. Removable prostheses are associated by the public with reduced oral comfort and poorer aesthetics [42]. It has also been shown that patient satisfaction diminishes significantly with the number of teeth remaining. Patients with at least 25 intact teeth are significantly more satisfied with any type of prosthesis than patients with 1 to 24 intact teeth [43]. Above all, however, patient-related factors are of importance: satisfaction with dentures is not only significantly dependent on personality type [44] but also on acceptance of tooth loss [45]. These patient-related factors often cannot be reliably determined in a clinical study. 

In the studies included, patient satisfaction was exclusively assessed by means of nonvalidated questionnaires that enquired about patients' subjective satisfaction. The oral health-related quality of life (OHQoL) instrument is a validated tool. OHQoL also diminishes considerably with the number of intact teeth (*P* < 0.001) and the condition of those remaining [46]. In patients with less than 20 intact teeth, it falls to half of the value for fully dentulous patients. Above all, the lack of the front teeth—even if these are replaced by a prosthesis—substantially lowers the OHQoL (*OR* = 21.5) [47]. However, demographic factors such as ethnicity or immigrant status have a stronger influence on the OHQoL than the status of the teeth [48]. Current studies even come to the conclusion that the OHQoL is not meaningful as an indicator for patient satisfaction or the disease burden of patients with tooth gaps [49]. 

### 4.4. Cleansability and Aftercare Required

The outcome “Denture cleansability and aftercare required” covers four different patient-related aspects: 

(1) effort involved in the cleaning of the remaining teeth, as well as (2) effort involved in the cleaning of the denture (the third and fourth aspects are described further below). 

The first and second aspects cover all measures demanded of the patient with regard to oral or prosthesis hygiene. Fixed and removable dentures differ fundamentally here, as the cleansability of the remaining teeth is considerably facilitated after the removable dentures are taken out, while extra effort is required for prosthesis hygiene. Fixed dentures are to be cleansed within the context required for routine denture care; not only the demands in time but also the dexterity required by the patient is greater, however, in comparison to the care of natural teeth. In patients no longer possessing sufficient motor capacities due to age or illness, fixed dentures are for this reason often contraindicated. The question as to whether and what influence the nature of the opposing dentition has on oral and prosthesis hygiene could not be answered in the present analysis. In principle it can, however, be assumed that for every denture, independently of the opposing arch, certain efforts are necessary for hygiene, efforts which are doubled, for example, if the opposing arch is equipped with a similar denture. 

Without taking opposing teeth into account, numerous studies have shown that, assuming adequate care, patients with fixed as well as those with removable dentures bear no increased risk of caries [50–52] or periodontitis [29, 53, 54]. For both fixed and removable dentures, adherence to specific regulations on design is essential in the production of the devices in order to keep plaque formation as low as possible and so ensure the hygienic qualities of the dentures [55]. In addition, it is the dentist's responsibility to inform the patient not only of the necessity and possibilities of oral and prosthesis hygiene, but also to motivate him or her on a regular basis [56]. The reality, however, is that 83% of all denture wearers are not properly informed and 12% of all wearers show no adequate behaviour regarding denture care [57]; in hospitalized patients the percentage is even 45% [58]. 

Further aspects of the outcome “Denture cleansability and aftercare required” are (3) the level of repair required for dentures and (4) the effort involved in denture aftercare. 

For denture repairs, not only the potential costs incurred but also the often necessary deprivation of the denture during repair in a dental laboratory are relevant issues for patients.

The following points were noted in the evaluation of the studies included.

1. To be able to draw robust conclusions, a randomized controlled design of the study is essential. This cannot, however, be easily ensured in studies on dental prostheses. Stratified randomization should be used, at least with regard to the different opposing dentition groups. The problem of randomized allocation of patients to the fixed or removable denture group arises from the fact that fixed dentures are more expensive to produce. The “split-mouth” study design used in numerous denture studies, that is, the intraindividual comparison between the left- and right-hand halves of the jaw of the same patient, is only suitable for the assessment of fixed dentures, not removable dentures, which extend over the whole jaw for one-sided tooth gaps.
2. In the case of non-RCTs, the intervention arches should be comparable. As an intact jaw has at least 14 teeth, not less than 2^14^ = 16,384 variations in tooth gaps are possible. To limit the number of subgroups, classification according to type, localization, and size of the gap, as practiced in this report, would be sensible, that is, specification of Kennedy class (I−IV) of the jaw, as well as gap width (number of missing teeth). By this classification the number of subgroups is reduced. These data should be supplemented by the number of intact teeth remaining, which should be similar between groups.
3. In the description of the opposing dentition, at least the five groups distinguished in this systematic review (ND, RPD, CD, FPD, and IFP) should also be assessed separately. In many publications, these data are only reported within the framework of the demographic description of the patient population, but the results are not presented separately for groups according to opposing dentition. In part, for example, the natural opposing dentition is treated as equal to a fixed denture in the opposing arch. In the case of a follow-up period of ten years or longer, it should be noted that, over time, the condition of the opposing dentition may change. Due to the loss of teeth in the opposing arch, natural opposing dentition may turn into residual dentition and can eventually lead to a fully edentulous arch. All these changes must be documented. It should further be considered whether only patients with a constant status of their opposing dentition should be included in the assessment in terms of a per-protocol (PP) analysis or whether, parallel to that, patients with changes in this status should be evaluated during the follow-up period by way of an intention-to-treat (ITT) analysis.
4. As a relevant comorbidity, the periodontal status of the remaining teeth should primarily be considered. Every type of denture—with the exception of implants—results in additional strain on the remaining teeth. If their stability is compromised by periodontal disease, this has an effect on the expected longevity of the denture. The presence of parafunctions such as bruxism is a second important factor, as dentures in people with such a condition are overstressed. Periodontitis and parafunctions should at least be adequately monitored.
5. Prognostic factors include smoking habits and oral hygiene. As these are changeable risk factors, smokers and patients with inadequate oral hygiene need not necessarily be excluded from the study, but could receive counseling for smoking cessation or be given instructions in oral hygiene. As the compliance of study participants is important in every long-term study, this would also represent a preselection with regard to less cooperative patients. Suitable variables as indicators of patients' oral hygiene are plaque and bleeding indices [30].
6. It is imperative that the prostheses are of uniform age. Many of the studies identified compared newly manufactured prostheses and others that had in part been used for over ten years. In prospective study designs, the age of the prostheses is equivalent to the follow-up period, which should not be subject to deviations. Furthermore, attention should be paid to a standardized manufacturing process of the prostheses. For fixed dentures this means the same preparation technique, the same molding material, the same alloy and ceramic material, and if possible the same dental laboratory. For removable dentures it would in principle be important to focus on a uniform, systematic design of the prosthesis, on the same materials, and on a standardized manufacturing process. Comparison of the level of repair required for differently fabricated dentures must be regarded as a methodological error in the study design. In retrospectively planned studies it can be assumed that the prostheses were not manufactured under standardized conditions.
7. As a matter of principle, only dental indications should be compared in which both fixed and removable dentures are considered as treatment options. For one- or two-sided free-end gaps (Kennedy class I+II) with a gap width of one tooth, a fixed denture in the form of an extension bridge is possible, but due to the greater strain arising through the leverage effect, the longevity or level of repair of such bridges is, for example, not comparable to that of conventional bridges in patients with Kennedy class III gaps [59]. Regarding the longevity of implants it should be noted that their survival rate depends, among other factors, on their position, length, diameter, and surface. If the jawbone material available is insufficient to fit an implant, then bone augmentation, that is, an increase in the local bone material, must first be performed. The survival rate of implants in augmented areas is again lower than that of those set in original, local bone material [60].
8. The assessment of outcomes must follow a standardized procedure. Although restrictions such as cost aspects, the impossibility of blinding, and the lack of randomization due to patient preferences play an important role in the conduct of dental care studies, some approximation to the state-of-the-art standards implemented in other areas of medical care would be desirable. Overcoming these restrictions represents a challenge that evidence-based dental medicine should successfully meet. Ultimately, the conduct of informative studies is urgently recommended in order to successfully clarify the present research questions.

## 5. Conclusions

This systematic review assesses the relevance of variations in the opposing dentition for the functionality of fixed and removable partial dentures. There is currently no proof of sufficient certainty regarding the relevance of opposing dentition in removable and fixed partial dentures for any of the following patient-relevant outcomes: “denture longevity,” “change in dietary habits,” “oral health-related QoL,” condensed into “patient satisfaction,” and “denture cleansability and aftercare required.” 

No evidence-based statements could be generated as to whether, and if so how, variations in the opposing dentition have a bearing on the decision to fit a partially edentulous arch with a fixed or removable partial denture. There were only few indications of increased patient satisfaction in favor of the fixed partial denture in combination with the opposing dentition variant of complete denture in the opposing arch. However, these indications are based on a small number of methodologically weak studies, and this is characteristic of the field of prosthetic dentistry, as the systematic review shows. 

## Figures and Tables

**Table 1:** Studies evaluated—summary.

Study	Indication	Intervention	Comparative intervention	Opposing dentition	Observation^a^	Setting (outpatient)
Randomized controlled trials						

Budtz-Jørgensen and Isidor, 1990 [12]	Partially edentulous mandible	Fixed partial denture	Removable partial denture	CD	1980-1981^b^ 1981–1986^c^	Aarhus, Denmark

Prospective interventional studies						

Balshi et al., 1996 [13]	Single-tooth gap in the molar area	Implant-supported crown Implant-supported bridge	—^d^	ND	n/a	Fort Washington, Pennsylvania, USA
						
Carlson and Yontchev, 1996 [14]	Residual dentition of mandibular canines	Fixed partial denture	—	CD	1973–1975^b^	Gothenburg, Sweden
						
Esquivel-Upshaw et al., 2008 [15]	Single-tooth gaps in posterior teeth	Fixed partial denture	—	ND	n/a	Florida, USA
						
Romeo et al., 2003 [16]	Partially edentulous patients	Implant-supported bridge	—	ND/CD/FPD/RPD	1994–2001	Milan, Italy
Ueda et al., 1993 [17]	Partially edentulous and edentulous patients	Implant-supported bridge	Implant-supported complete denture	ND/RPD/IFP	n/a	Nagoya, Japan
Wayler et al., 1984 [18]	Dentures and Intact dentition	Partial denture	Complete dentures and intact dentition	ND/CD/RPD	n/a	Boston, USA

Retrospective interventional studies						

Randow et al., 1986 [19]	Partially edentulous	Fixed partial denture	—	ND/CD/FPD/RPD	1974–1976^b^ 1981–1983^c^	Malmö, Sweden
						
Studer et al., 1998 [20]	Partially edentulous patients	Fixed-removable reconstruction	—	ND/CD/FPD/RPD	1976–1993^b^	Zurich, Switzerland
						
Yli-Urpo et al.,1985[21]	Removable dentures in need of repair	Removable partial denture	Removable partial denture	ND/CD/RPD	1978–1983^b^ 1983-1984^e^	Kuopio, Finland
						

Cross-sectional studies/questionnaires^f^						

Frank et al.,1998 [22]	Mandibular partial dentures	Partial denture	—	ND/CD/FPD/RPD	1990–1995^b^	Seattle, Washington, USA
Hummel et al., 2002 [23]	Partial dentures	Partial denture	—	ND/CD/FPD/RPD	1988–1994	Overall pop., USA
Lassila et al., 1985 [24]	Partial and complete dentures	Partial denture	Complete denture	ND/CD/RPD	n/a	Turku, Finland
Liedberg et al., 2005 [25]	(Complete) dentures, intact dentition	Partial denture	Complete denture and intact dentition	ND/CD/RPD	1985–1987	Malmö, Sweden
Ow et al., 1997 [26]	Dentures, intact dentition	Partial denture	Complete denture and intact dentition	ND	n/a	Singapore
Tuominen et al., 1989 [27]	Partial dentures, intact dentition	Partial denture	Intact dentition	ND/FPD/RPD	n/a	Overall pop., Finland
Vallittu et al., 1993 [28]	Removable dentures in need of repair	Removable partial denture	Removable partial denture	ND/CD/FPD/RPD	n/a	Lathi/Kuopio, Finland

^a^ The exact periods of observation, which may differ from the period the study was conducted, are listed separately in the summary tables.

^b^Manufacture of prostheses.

^c^Followup.

^d^If not specified, there is no comparative intervention or no intervention evaluated.

^e^Repair of prostheses.

^f^Noninterventional inquiries by questionnaire.

CD: complete denture, FPD: fixed partial denture, IFP: implant-supported fixed prostheses, ND: natural dentition, RPD: removable partial denture, n/a: not available.

**Table 2:** Description of patient population.

Study	Gender m/f	Mean age (range)	Number of patients screened	Number of patients with intervention	Patients evaluated	Drop-outs *n* (%)
Balshi et al., 1996 [13]	15/29^a^	n/a (n/a)	45	45	45	0
Isidor and Budtz-Jørgensen, 1990 [29]	25/28	69.0 (61–83)	53	53	43	10 (23%)
Carlson and Yontchev, 1996 [14]	11/1	53 (33–74)	12	12	8	4 (33.3%)
Esquivel-Upshaw et al., 2008 [15]	3/18	n/a (30–62)	21	21	21	0
Frank et al., 1998 [22]	402/398	59 (n/a)	800	410	410	—^b^
Hummel et al., 2002 [23]	n/a	n/a (17–n/a)	17884	1306	1303	—^b^
Lassila et al., 1985 [24]	47/42	62.5 (n/a)	89	89	89	—^b^
Liedberg et al., 2005 [25]	483/0	68 (67-68)	483	483	474	—^b^
Ow et al., 1997 [26]	312/579	65.9 (55–91)	891	891	871	—^b^
Randow et al., 1986 [19]	96/145^c^	51.6 (n/a)^c^	281	267	241	26 (11%)
Romeo et al., 2003 [16]	16/22	51 (21–71)	38	38	38	0
Studer et al., 1998 [20]	46/66	57.7 (28–84)	155	155	112	43 (38%)
Tuominen et al., 1989 [27]	2568/2460	51.9 (30–n/a)	5028	5028	5028	—^b^
Ueda et al., 1993 [17]	11/11	54.4 (34–74)	22	22	22	0
Vallittu et al., 1993 [28]	n/a	n/a (n/a)	—	266	266	—^b^
Wayler et al., 1984 [18]	1221/0	50.5 (25–79)	1221	1133	1133	88 (8%)
Yli-Urpo et al., 1985 [21]	62/60	n/a (n/a)	—	122	122	0

^a^No information on the gender of 3 patients.

^b^There were no drop-outs with one-time data collection, though cross-sectional studies contain no information on response rates.

^c^Data applies to evaluated patients; all other apply to included patients.

f: female, m: male, n: number of patients, n/a: not available.

**Table 3:** Quality of studies and publications.

Study	Random group assignment	Patient recruitment	<20% drop-outs/reasons given	Age of dentures^a^	Consistency of data within publication	Presentation of results	Biometric quality^b^
Randomized controlled trials							

Budtz-Jørgensen and Isidor, 1990 [12]	Unclear	Consecutive	yes/yes	Homogenous	Yes	Complete	Minor flaws^c^

Prospective interventional studies							

Balshi et al., 1996 [13]	—	Consecutive	No drop-outs^d^	Homogeneous	Yes	Incomplete	Minor flaws
Carlson and Yontchev, 1996 [14]	—	Unclear	No/yes	Inhomogeneous	Yes	Complete	Major flaws
Esquivel-Upshaw et al., 2008 [15]	—	Unclear	No drop-outs^d^	Homogeneous	Yes	Incomplete	Minor flaws
Romeo et al., 2003 [16]	—	Consecutive	No drop-outs^d^	Inhomogeneous	Yes	Incomplete	Major flaws
Ueda et al., 1993 [17]	—	Consecutive	No drop-outs^d^	Inhomogeneous	Yes	Incomplete	Major flaws
Wayler et al., 1984 [18]	—	Unclear	Yes/no	n/a	No	Incomplete	Major flaws

Retrospective interventional studies							

Randow et al., 1986 [19]	—	Unclear	Yes/no	Homogeneous	Yes	Incomplete	Major flaws
Studer et al., 1998 [20]	—	Consecutive	No/yes	Inhomogeneous	Yes	Incomplete	Major flaws
Yli-Urpo et al., 1985 [21]	—	Consecutive	No drop-outs^d^	Inhomogeneous	No	Incomplete	Major flaws

Cross-sectional studies (questionnaires)^e^							

Frank et al., 1998 [22]	—	Unclear	—^f^	Inhomogeneous	Yes	Incomplete	Major flaws
Hummel et al., 2002 [23]	—	Cross-sectional	—^f^	n/a	Yes	Incomplete	Major flaws
Lassila et al., 1985 [24]	—	Unclear	—^f^	Inhomogeneous	Yes	Incomplete	Major flaws
Liedberg et al., 2005 [25]	—	Cohort study	—^f^	Inhomogeneous	Yes	Incomplete	Major flaws
Ow et al., 1997 [26]	—	Random sample	—^f^	n/a	Yes	Incomplete	Major flaws
Tuominen et al., 1989 [27]	—	Random sample	—^f^	n/a	Yes	Incomplete	Major flaws
Vallittu et al.,1993 [28]	—	Unclear	—^f^	n/a	No	Incomplete	Major flaws

^a^The homogeneity or inhomogeneity of the age of the prosthesis as a qualitative statement of the authors of the report refers to the range, where it is stated.

^b^The biometric quality concerns the aspects of the study that are linked to the question, not the whole of the study as such.

^c^The assignment was done on the basis of X-ray adjusting for other patient characteristics, and therefore the biometric quality was affected.

^d^No drop-outs were observed in the study, irrespective of whether they occurred.

^e^The approach with one-time inquiries without followup is similar to a cross-sectional design.

^f^The studies do not give response rates for one-time inquiries.

n/a: not available.

**Table 4:** Longevity of fixed partial dentures.

Study	Intervention	Kennedy Class	Gap width/residual dentition^a^	Opposing dentition	Followup in month (Range)	Success rate (%)^b^
Randomized controlled trials						

Budtz-Jørgensen and Isidor, 1990 [12]	FPD	n/a	RD = 6.9 ± 1.7 GW = 2 − 3 (44.4%) GW = 4 − 5 (25.9%) GW = 9 − 11 (29.7%)	CD	60 ± 0	95.2%

Prospective interventional studies						

Balshi et al., 1996 [13]	IFP	III	GW = 1	ND	36 ± 0	97.8%
Carlson and Yontchev, 1996 [14]	FPD	I	RD = 2 ± 0	CD	n/a (36–180)	50.0%
Esquivel-Upshaw et al., 2008 [15]	FPD	III	GW = 1	ND	48 ± 0	86.7%
						
Romeo et al., 2003 [16]	IFP	I (19.5%) II (80.5%)	GW = 2 (20.8%) GW = 3 (60.4%) GW = 4 (12.5%) GW = 5 (6.3%)	ND/FPD (76%) IFP (24%)	44.5 ± 235 (12–84)	97.2% 100%
Ueda et al., 1993 [17]	IFP	I (21.4%) II (42.9%) III (7.1%) IV (28.6%)	n/a	ND (37,0%)^b^ RPD (29,6%)^b^ IFP (33,4%)^b^	n/a (6.8–10)	96.2%^c^

Retrospective interventional studies						

Randow et al., 1986 [19]	FPD	I/II (71.2%) III (28.8%)	n/a	ND/FPD (54.7%) RPD/CD (16.5%) ND/FPD (28.8%)	84 ± 0	75.6% 78.7% 92.7%

^a^States number of missing teeth in the respective gap, not the number of patients, as these can show several teeth gaps.

^b^Percentage of manufactured prostheses, that were still functional after the defined observation period.

^c^Data given only for partially edentulous and edentulous patients combined.

CD: complete denture, FPD: fixed partial denture, GW: gap width, IFP: implant-supported fixed prostheses, ND: natural dentition, RD: residual dentition, RPD: removable partial denture, n/a: not available.

**Table 5:** Longevity of removable denture.

Study	Intervention	Kennedy Class	Gap width/residual dentition	Opposing dentition	Followup in month (range)	Success rate (%)^a^
Randomized controlled trials						

Budtz-Jørgensen and Isidor, 1990 [12]	RPD	n/a	RD = 7.5 ± 1.7	CD	60 ± 0	100%

Retrospective interventional studies						

Studer et al., 1998 [20]	RPD	I (63.1%) II (23.8%) III (10.8%) IV (2.3%)	n/a	ND/FPD (36.9%) RPD (47.7%) CD (15.4%)	66 ± 42.9 (12–192)	61.5%

^a^Percentage of manufactured prostheses, that were still functional after the defined observation period.

CD: complete denture, FPD: fixed partial denture, IFP: implant-supported fixed prostheses, ND: natural dentition, RD: residual dentition, RPD: removable partial denture, n/a: not available.

**Table 6:** Results on the longevity of fixed and removable prostheses.

Interventions	Fixed prostheses	Removable prostheses
Studies	7	2

RCT	1	1
Prospective	5	0
Retrospective	1	1
Questionnaire	0	0

No flaws	1	0
Minor flaws	2	1
Major flaws	4	1

Patient age (a)	55.8 [83,4%^b^]	63.4 [100%^b^]
Gender (% m)	45.6 [100%^b^]	44.2 [100%^b^]

Oral hygiene index^a^	0.4–1.0 [5.8%^b^]	0.4–1.0 [16.7%^b^]
Tobacco consumption	0 [10.4%^b^]	—

Number of prostheses	463	156
Followup (Mo)	65.9 [100%^b^]	95.0 [100%^b^]
Drop-outs (%)	7.7 [100%^b^]	26.9 [100%^b^]

Residual dentition	8.7 [14.9%^b^]	7.5 [16.7%^b^]
Width of gaps	0.9 [24.5%^b^]	—
Kennedy I	7.1%	52.6%
Kennedy II	7.8%	19.8%
Kennedy III	34.6%	9.0%
Kennedy IV	0.9%	1.9%
Kennedy n.a.^c^	49.6%	16.7%

Opposing arch ND	16.6%	0%
Opposing arch FPD	2.8%	0%
Opposing arch RPD	0%	39.7%
Opposing arch CD	8.4%	29.5%
Opposing arch n.a.^c^	72.2%	30.8%

Functional capability with ND in OA (%)	86.7^d^ for FPD {48^e^} (Esquivel-Upshaw et al., 2008 [15])
	97.8^d^ for IFP {36^e^} (Balshi et al., 1996 [13])

Functional capability with ND in OA (%)	95.2^d^ for FPD {60^e^} (Budtz-Jørgensen and Isidor, 1990 [12])	100^d^{60^e^} (Budtz-Jørgensen and Isidor, 1990 [12])

Functional capability with ND in OA (%)	100^d^ for IFP {44.5^e^} (Romeo et al., 2003 [16])*	

^a^Silness and Löe plaque index [30].

^b^Percentage of the sample to which results refer to.

^c^Data in publication could not be evaluated, since several groups were pooled or could not be itemized.

^d^Percentage of functional prostheses.

^e^(Intermediate) followup in month.

*Results from study with major biometrical flaws.

CD: complete denture, FPD: fixed partial denture, IFP: implant-supported fixed denture, m: male, mo: months, ND: natural dentition, OA: opposing arch, RCT: randomized controlled trial, RPD: removable partial denture, n/a: not available.

**Table 7:** Effect of fixed denture on eating habits.

Study	Question	Kennedy Class	Gap width/ Residual dentition	Opposing dentition	Age of prosthesis in month (range)	Result
Prospective interventional trials						

Ueda et al., 1993 [17]	Masticatory efficiency improvement by the use of implants?	I (21.4%) II (42.9%) III (7.1%) IV (28.6%)		ND (37.0%)^a^		+39.5^a,^ ^b^
			n/a	RPD (29.6%)^a^	n/a (6.8–10)	+48.8^a,^ ^b^
				IFP (33.4%)^a^		+62.5^a,^ ^b^

^a^Data given only for partially edentulous and edentulous patients combined.

^b^Rating system: for each of the 20 Japanese test meals (Bean curd, Boiled rice with tea, Noodles, Pudding, Lettuce, Shrimp tempura, Sliced cucumber, Boiled fish paste, Tender steak, Pickled radish, Herring roe, Cookie, Cracker, Rice cake cubes, Sliced raw cuttlefish, Salami, Dried cuttlefish, Chewing gum, Biting on an apple, and Biting off a cotton thread) 5 points are allocated if the patient is able to chew it (100 points maximum). The average difference between preoperative and postoperative examination was calculated (positive values represent a better postoperative result).

IFP: implant-supported fixed prostheses, ND: natural dentition, RPD: removable partial denture, n/a: not available.

**Table 8:** Effect of removable denture on eating habits.

Study	Question	Kennedy Class	Gap width/residual dentition	Opposing dentition	Age of prosthesis in month (range)	Result
Prospective interventional trials						

				ND (66%)		1.35^a^
Wayler et al., 1984 [18]	Is the test food easy to swallow?	n/a	n/a	RPD (19%)	n/a	1.42^a^
				CD (15%)		1.56^a^

One-time data collection studies/questionnaires						

Frank et al., 1998 [22]	Do you use your lower partial denture for eating?	I (58%) II (21%) III (13%) IV (6%) n/a (2%)	n/a	ND (18%) FPD (19%) RPD (25%) CD (36%) n/a (2%)	n/a (12–180)	85.9%^b^
	Do you have difficulties in chewing with your lower partial denture?					42.1%^b^
	Does your lower partial denture limit your choice of foods?					46.2%^b^
	Does your lower partial denture affect the taste of food?					15.6%^b^
	Does food get under your lower partial denture when you eat?					83.8%^b^
						
Liedberg et al., 2005 [25]	How often do you eat hard food?	n/a	RD = 17.4^c^ RD = 11.8^c^ RD = 5.0^c^	ND (37%) RPD (19%) CD (44%)	n/a	98.9%^d^ 100.1%^d^ 100.1%^d^
	How often do you eat soft food?		RD = 17.4^c^ RD = 11.8^c^ RD = 5.0^c^	ND (37%) RPD (19%) CD (44%)		123.7%^d^ 124.0%^d^ 122.4%^d^
						
Ow et al., 1997 [26]	Do you face chewing problems with your prosthesis?	n/a	n/a	ND	n/a	8%^b^

^a^Rating scheme from 1 (positive) to 4 (negative) with 13 test foods (Hard rolls, French or Italian bread, Pot roast, Steak, Salami, Fried clams, Fried chicken, Raw carrots, Celery, Cole slaw, Cucumbers, Apples, and Peanuts), mean values calculated per patient and group, no variance given.

^b^Percentage of patients answering “yes” to the question.

^c^No variance given.

^d^How often and how much hard (Pork, Beef, Raw Vegetables, Apples, Pears, Wholemeal bread, and Crisp bread) and soft (Cod-fish, Herring, Minced meat, Boiled vegetables, Sausages, and Bananas) food was eaten in a month. The eating habits of the participant showing the least impairment in instrumental measurement of masticatory efficiency were defined as 100%.

CD: complete denture, FPD: fixed partial denture, IFP: implant-supported fixed prostheses, ND: natural dentition, RD: residual dentition, RPD: removable partial denture, n/a: not available.

**Table 9:** Results on the eating habits of fixed and removable prostheses.

Interventions	Fixed prostheses	Removable prostheses
Studies	1	4

RCT	0	0
Prospective	1	1
Retrospective	0	0
Questionnaire	0	3

No flaws	0	0
Minor flaws	0	0
Major flaws	1	4

Patient age (a)	54.4 [100%]	60.9 [100%]
Gender (% m)	50.0 [100%]	71.3 [100%]

Number of prostheses	14	1004
Followup (Mo)	8.4 [100%]	—
Drop-outs (%)	0 [100%]	—

Residual dentition	—	11.4 [13,0%]
Width of gaps	—	—
Kennedy I	21.4%	23.8%
Kennedy II	42.9%	8.8%
Kennedy III	7.1%	5.3%
Kennedy IV	28.6%	2.3%
Kennedy n/a^a^	0%	59.9%

Opposing arch ND	0%	50.5%
Opposing arch FPD	0%	7.7%
Opposing arch RPD	0%	17.0%
Opposing arch CD	0%	24.0%
Opposing dentition n/a^a^	100%	0.8%

Hard food with ND in opposing arch		98.9^b^{n/a^c^} (Liedberg et al., 2005 [25])*

Hard food with RPD in opposing arch		100.1^b^{n/a^c^} (Liedberg et al., 2005 [25]) *

Hard food with CD in opposing arch		100.1^b^{n/a^c^} (Liedberg et al., 2005 [25])*

Soft food with ND in opposing arch		123.7^b^{n/a^c^} (Liedberg et al., 2005 [25])*

Soft food with RPD in opposing arch		124.0^b^{n/a^c^} (Liedberg et al., 2005 [25])*

Soft food with CD in opposing arch		122.4^b^{n/a^c^} (Liedberg et al., 2005 [25])*

Chewing problems with ND in opposing arch		8^d^{n/a^c^} (Ow et al., 1997 [26])*

^a^Data in publication not given or could not be evaluated, since several groups were pooled or could not be itemized.

^b^Frequency of intake measured with 12 test foods in percent relatively to normal value.

^c^(Intermediate) followup in month.

^d^Percentage of patients which stated masticatory problems with prostheses.

*Results of study with major biometric flaws.

CD: complete denture, FPD: fixed partial denture m: male, mo: month, ND: natural dentition, OA: opposing arch, RCT: randomized controlled study, RPD: removable partial dentures.

## References

[1] Bundesausschuss G (2010). Festzuschuss Richtlinie: Gegenbezahlung bei der Versorgung mit festsitzendem Zahnersatz. *Bundesanzeiger*.

[2] Kerschbaum T (2006). Zahnverlust und prothetische Versorgung. *Vierte Deutsche Mundgesundheitsstudie (DMS IV): neue Ergebnisse zu oralen Erkrankungsprävalenzen, Risikogruppen und zum zahnärztlichen Versorgungsgrad in Deutschland*.

[3] Plotnick IJ, Beresin VE, Simkins AB (1975). The effects of variations in the opposing dentition on changes in the partially edentulous mandible. Part III. Tooth mobility and chewing efficiency with various maxillary dentitions. *The Journal of Prosthetic Dentistry*.

[4] McGarry TJ, Nimmo A, Skiba JF (2002). Classification system for partial edentulism. *Journal of Prosthodontics*.

[5] Osterberg T, Hedegård B, Säter G (1984). Variation in dental health in 70-year old men and women in Göteborg, Sweden. A cross-sectional epidemiological study including longitudinal and cohort effects. *Swedish Dental Journal*.

[6] Bain CA, Weng D, Meltzer A, Kohles SS, Stach RM (708). A meta-analysis evaluating the risk for implant failure in patients who smoke. *Compendium of Continuing Education in Dentistry*.

[7] Mazurat RD (1992). Longevity of partial, complete and fixed prostheses: a literature review. *Journal of Canadian Dental Association*.

[8] Walls AWG, Steele JG (2004). The relationship between oral health and nutrition in older people. *Mechanisms of Ageing and Development*.

[9] Boretti G, Bickel M, Geering AH (1995). A review of masticatory ability and efficiency. *The Journal of Prosthetic Dentistry*.

[10] Shay K (2000). Denture hygiene: a review and update. *The Journal of Contemporary Dental Practice*.

[11] Kennedy E (1928). Partial denture construction. *Dent Items Interest*.

[12] Budtz-Jørgensen E, Isidor F (1990). A 5-year longitudinal study of cantilevered fixed partial dentures compared with removable partial dentures in a geriatric population. *The Journal of Prosthetic Dentistry*.

[13] Balshi TJ, Hernandez RE, Pryszlak MC, Rangert B (1996). A comparative study of one implant versus two replacing a single molar. *International Journal of Oral and Maxillofacial Implants*.

[14] Carlson BR, Yontchev E (1996). Long-term observations of extensive fixed partial dentures on mandibular canine teeth. *Journal of Oral Rehabilitation*.

[15] Esquivel-Upshaw JF, Young H, Jones J, Yang M, Anusavice KJ (2008). Four-year clinical performance of a lithia disilicate-based core ceramic for posterior fixed partial dentures. *International Journal of Prosthodontics*.

[16] Romeo E, Lops D, Margutti E, Ghisolfi M, Chiapasco M, Vogel G (2003). Implant-supported fixed cantilever prostheses in partially edentulous arches. A seven-year prospective study. *Clinical Oral Implants Research*.

[17] Ueda M, Niimi A, Murakami I, Kaneda T (1993). Masticatory improvement using osseointegrated implants: analysis of Japanese patients’ responses through questionnaires. *The International Journal of Oral & Maxillofacial Implants*.

[18] Wayler AH, Muench ME, Kapur KK, Chauncey HH (1984). Masticatory performance and food acceptability in persons with removable partial dentures, full dentures and intact natural dentition. *Journals of Gerontology*.

[19] Randow K, Glantz PO, Zöger B (1986). Technical failures and some related clinical complications in extensive fixed prosthodontics. An epidemiological study of long-term clinical quality. *Acta Odontologica Scandinavica*.

[20] Studer SP, Mäder C, Stahel W, Schärer P (1998). A retrospective study of combined fixed-removable reconstructions with their analysis of failures. *Journal of Oral Rehabilitation*.

[21] Yli-Urpo A, Lappalainen R, Huuskonen O (1985). Frequency of damage to and need for repairs of removable dentures. *Proceedings of the Finnish Dental Society*.

[22] Frank RP, Milgrom P, Leroux BG, Hawkins NR (1998). Treatment outcomes with mandibular removable partial dentures: a population-based study of patient satisfaction. *The Journal of Prosthetic Dentistry*.

[23] Hummel SK, Wilson MA, Marker VA, Nunn ME (2002). Quality of removable partial dentures worn by the adult U.S. population. *The Journal of Prosthetic Dentistry*.

[24] Lassila V, Holmlund I, Koivumaa KK (1985). Bite force and its correlations in different denture types. *Acta Odontologica Scandinavica*.

[25] Liedberg B, Stoltze K, Owall B (2005). The masticatory handicap of wearing removable dentures in elderly men. *Gerodontology*.

[26] Ow RKK, Loh T, Neo J, Khoo J (1997). Perceived masticatory function among elderly people. *Journal of Oral Rehabilitation*.

[27] Tuominen R, Ranta K, Paunio I (1989). Wearing of removable partial dentures in relation to periodontal pockets. *Journal of Oral Rehabilitation*.

[28] Vallittu PK, Lassila VP, Lappalainen R (1993). Evaluation of damage to removable dentures in two cities in Finland. *Acta Odontologica Scandinavica*.

[29] Isidor F, Budtz-Jørgensen E (1990). Periodontal conditions following treatment with distally extending cantilever bridges or removable partial dentures in elderly patients. A 5-year study. *Journal of Periodontology*.

[30] Silness J, Löe H (1964). Periodontal disease in pregnancy; II: correlation between oral hygiene and periodontal condition. *Acta Odontologica Scandinavica*.

[31] Walton JN, Gardner FM, Agar JR (1986). A survey of crown and fixed partial denture failures: length of service and reasons for replacement. *The Journal of Prosthetic Dentistry*.

[32] Wetherell JD, Smales RJ (1980). Partial denture failures: a long-term clinical survey. *Journal of Dentistry*.

[33] Albrektsson T, Lekholm U (1989). Osseointegration: current state of the art. *Dental Clinics of North America*.

[34] Gunne HS (1985). The effect of removable partial dentures on mastication and dietary intake. *Acta odontologica Scandinavica*.

[35] Budtz-Jørgensen E, Chung JP, Mojon P (2000). Successful aging—the case for prosthetic therapy. *Journal of Public Health Dentistry*.

[36] Liedberg B, Norlén P, Owall B, Stoltze K (2004). Masticatory and nutritional aspects on fixed and removable partial dentures. *Clinical Oral Investigations*.

[37] Vinton P, Manly RS (1955). Masticatory efficiency during the period of adjustment to dentures. *The Journal of Prosthetic Dentistry*.

[38] Agerberg G, Carlsson GE (1981). Chewing ability in relation to dental and general health. Analyses of data obtained from a questionnaire. *Acta Odontologica Scandinavica*.

[39] Carlsson GE, Persson G (1967). Morphologic changes of the mandible after extraction and wearing of dentures. A longitudinal, clinical, and x-ray cephalometric study covering 5 years. *Odontologisk Revy*.

[40] Tan K, Li AZJ, Chan ESY (2005). Patient satisfaction with fixed partial dentures: a 5-year retrospective study. *Singapore Dental Journal*.

[41] Čelebić A, Knezović-Zlatarić D (2003). A comparison of patient’s satisfaction between complete and partial removable denture wearers. *Journal of Dentistry*.

[42] Witter DJ, van Elteren P, Käyser AF, van Rossum MJ (1989). The effect of removable partial dentures on the oral function in shortened dental arches. *Journal of Oral Rehabilitation*.

[43] Jones JA, Orner MB, Spiro A, Kressin NR (2003). Tooth loss and dentures: patients’ perspectives. *International Dental Journal*.

[44] Özdemir AK, Özdemir HD, Polat NT, Turgut M, Sezer H (2006). The effect of personality type on denture satisfaction. *International Journal of Prosthodontics*.

[45] Wong MCM, McMillan AS (2005). Tooth loss, denture wearing and oral health-related quality of life in elderly Chinese people. *Community Dental Health*.

[46] McGrath C, Bedi R (2001). Can dentures improve the quality of life of those who have experienced considerable tooth loss?. *Journal of Dentistry*.

[47] Walter MH, Woronuk JI, Tan HK (2007). Oral health related quality of life and its association with sociodemographic and clinical findings in 3 northern outreach clinics. *Journal of the Canadian Dental Association*.

[48] Swoboda J, Kiyak HA, Persson RE (2006). Predictors of oral health quality of life in older adults. *Special Care in Dentistry*.

[49] Jones JA, Kressin NR, Kazis LE (2006). Oral conditions and quality of life. *Journal of Ambulatory Care Management*.

[50] Bergman B, Hugoson A, Olsson CO (1982). Caries, periodontal and prosthetic findings in patients with removable partial dentures: a ten-year longitudinal study. *The Journal of Prosthetic Dentistry*.

[51] Tuominen R, Ranta K, Paunio I (1988). Wearing of removable partial dentures in relation to dental caries. *Journal of Oral Rehabilitation*.

[52] Lappalainen R, Koskenranta-Wuorinen P, Markkanen H (1987). Periodontal and cariological status in relation to different combinations of removable dentures in elderly men. *Gerodontics*.

[53] Brill N, Tryde G, Stoltze K, El Ghamrawy EA (1977). Ecologic changes in the oral cavity caused by removable partial dentures. *The Journal of Prosthetic Dentistry*.

[54] Stipho HD, Murphy WM, Adams D (1978). Effect of oral prostheses on plaque accumulation. *British Dental Journal*.

[55] Öwall B, Budtz-Jörgensen E, Davenport J (2002). Removable partial denture design: a need to focus on hygienic principles?. *International Journal of Prosthodontics*.

[56] Budtz-Jørgensen E (1979). Materials and methods for cleaning dentures. *The Journal of Prosthetic Dentistry*.

[57] Dikbas I, Koksal T, Calikkocaoglu S (2006). Investigation of the cleanliness of dentures in a university hospital. *International Journal of Prosthodontics*.

[58] Peltola P, Vehkalahti MM, Simoila R (2007). Effects of 11-month interventions on oral cleanliness among the long-term hospitalised elderly. *Gerodontology*.

[59] Pjetursson BE, Tan K, Lang NP, Chan ESY (2004). A systematic review of the survival and complication rates of fixed partial dentures (FPDs) after an observation period of at least 5 years III. Conventional FPDs. *Clinical Oral Implants Research*.

[60] Graziani F, Donos N, Needleman I, Gabriele M, Tonetti M (2004). Comparison of implant survival following sinus floor augmentation procedures with implants placed in pristine posterior maxillary bone: a systematic review. *Clinical Oral Implants Research*.

